# Impedance spectroscopy data of Ag_x_(Ge_16_Sb_12_Se_72_)_100-x_ chalcogenide glasses

**DOI:** 10.1016/j.dib.2019.01.025

**Published:** 2019-01-18

**Authors:** Deepak S. Patil, Manisha Konale, Tomas Wagner

**Affiliations:** a Department of Chemistry, New Mexico Highlands University, Las Vegas, NM 87701, USA; b School of Mechanical & Materials Engineering, Washington State University, Pullman, WA 99164, USA; c Department of General and Inorganic Chemistry, Faculty of Chemical Technology, University of Pardubice, Studentska 95, Pardubice 53210, Czech Republic

**Keywords:** Impedance spectroscopy, Ionic conductivity, Random-walk model, Chalcogenide glasses

## Abstract

Impedance spectroscopy is a valuable tool for the analysis of the ionic conductivity of both solid and liquid state materials. Chalcogenide glasses are well known for their high ionic conductivity nature and wide compositional flexibility. As the GeSbSe material has high glass forming ability, it is expected that the materials can be doped with a high amount of foreign element (in the present case Ag). For all of these reasons, the GeSbSe materials can be expected as a potential candidate for solid state electrolyte for ionic batteries. The ionic conductivity behavior of Ag_x_(Ge_16_Sb_12_Se_72_)_100-x_ chalcogenide glasses were studied using impedance a primary tool.

In the present article, you will find the impedance data of Ag_x_(Ge_16_Sb_12_Se_72_)_100-x_ chalcogenide glass system. From the impedance data, real and imaginary parts of conductivities were extracted and plotted as a function of applied frequency. The interpretation of the current article data were given in “Percolation behavior of Ag in Ge16Sb12Se72 glassy matrix and its impact on corresponding ionic conductivity” [1].

**Specifications table**

**Table:** 

Subject area	*Physics, chemistry, materials science*
More specific subject area	*Solid state electrolyte, Ionic Conductivity, Impedance Spectroscopy*
Type of data	*Table, image, figure*
How data was acquired	*Impedance Spectroscopy on Metrohm Autolab instrument with model name PGSTAT 302 N with the FRA32 and NOVA 10 software*
Data format	*Raw, analyzed*
Experimental factors	*Samples were cut into pellets, polished and sputtered with gold on both sides of pellets for good electrical contacts*
Experimental features	*All samples were prepared using conventional melt-quench method. Impedance spectroscopic measurements were carried out with an applied input voltage of 0.1 V, the temperature range from 295 K to 368 K, and a frequency range from 1 Hz to 50 kHz. The real and imaginary part of conductivities are extracted using random-walk model*[2]
Data source location	*Institute of Chemical Sciences of Rennes UMR CNRS 6226, University of Rennes 1, Beaulieu Campus, 35042 Rennes, France*
Data accessibility	*Data is presented in this article and*https://data.mendeley.com/datasets/k34nr3tm8r/draft?a=86262b74-1bf7-4e68-b28e-38943d9bd081
Related research article	D.S. Patil, M. Konale, S. Cozic, L. Calvez, V. Zima, T. Wagner, J.S. McCloy, D. Le Coq, Percolation behavior of Ag in Ge16Sb12Se72 glassy matrix and its impact on corresponding ionic conductivity, Journal of Alloys and Compounds, 782 (2019) 375–383 [1].

**Value of the data**

• The current impedance data is very useful for comparing similar ion conducting disordered materials.
• The random-walk model data fitting to experimental values shows an importance of random-walk model.
• The data shows influence of Ag doping concentrations on impedance behavior.
• It also shows the behavior of conductivity with respect to temperature.
• The current data shows the importance of impedance spectroscopy and random-walk model to get more depth information about ionic conduction in disordered materials.

## 1 Data

**Fig. 1:**
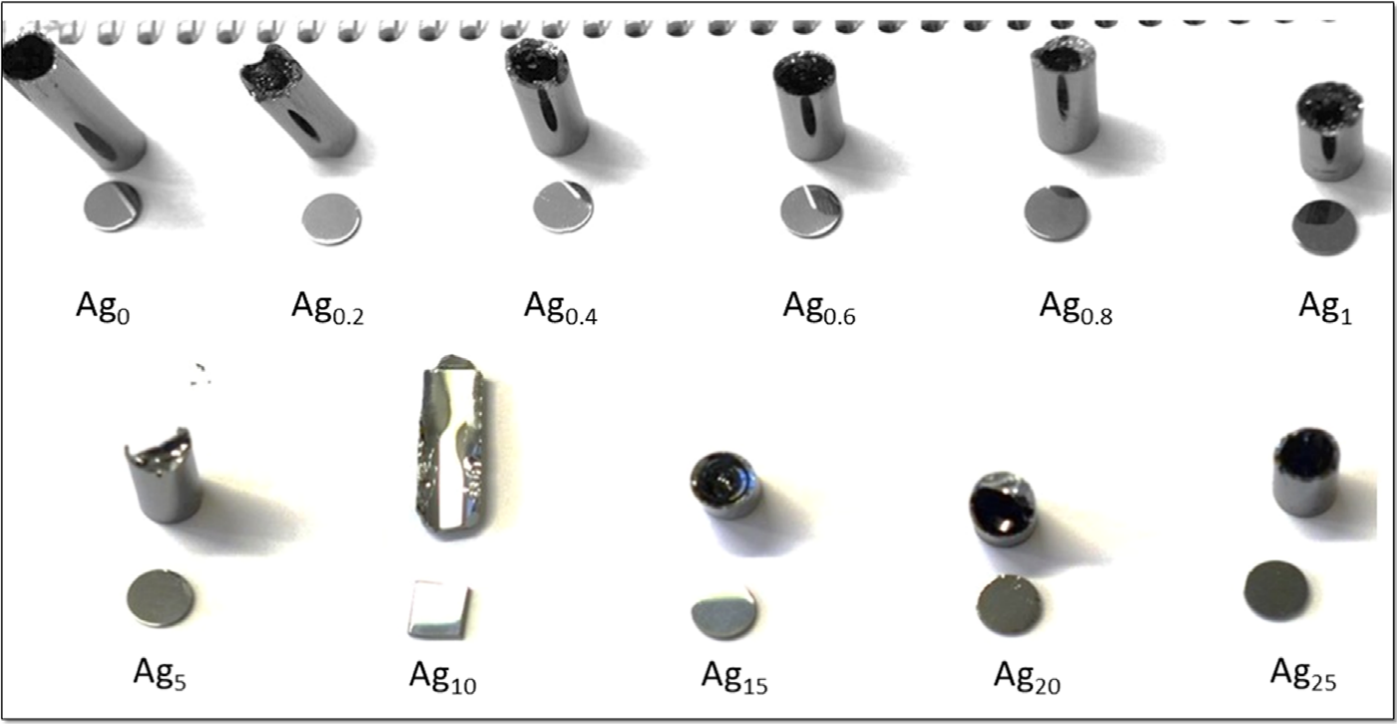
Images of all prepared glasses Ag_x_(Ge_16_Sb_12_Se_72_)_100-x_ with the polished disks prepared from corresponding glass rod.

**Fig. 2:**
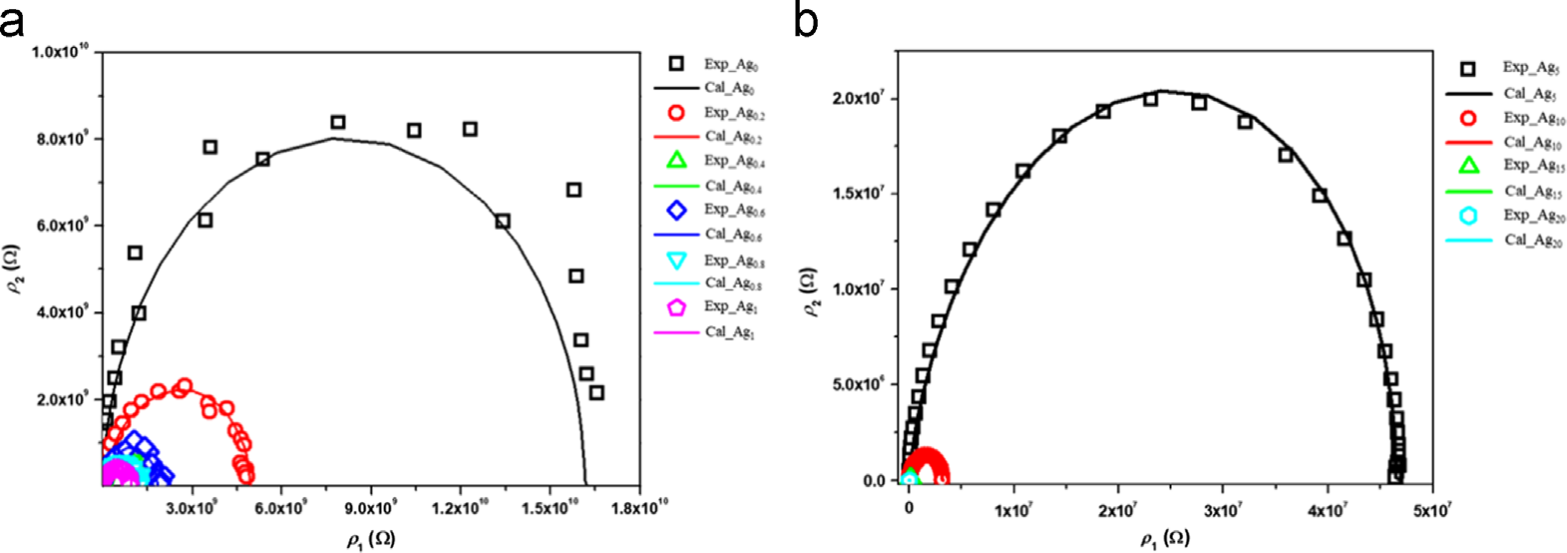
a) and b) are the nyquist plots of real (*ρ*_1_) and imaginary (*ρ*_2_) part of resistivity response from Ag0-Ag1 and Ag5-Ag20 set of samples respectively at 368 K. The open symbols represent the experimental values whereas solid line represents the random-walk model fitting.

**Fig. 3:**
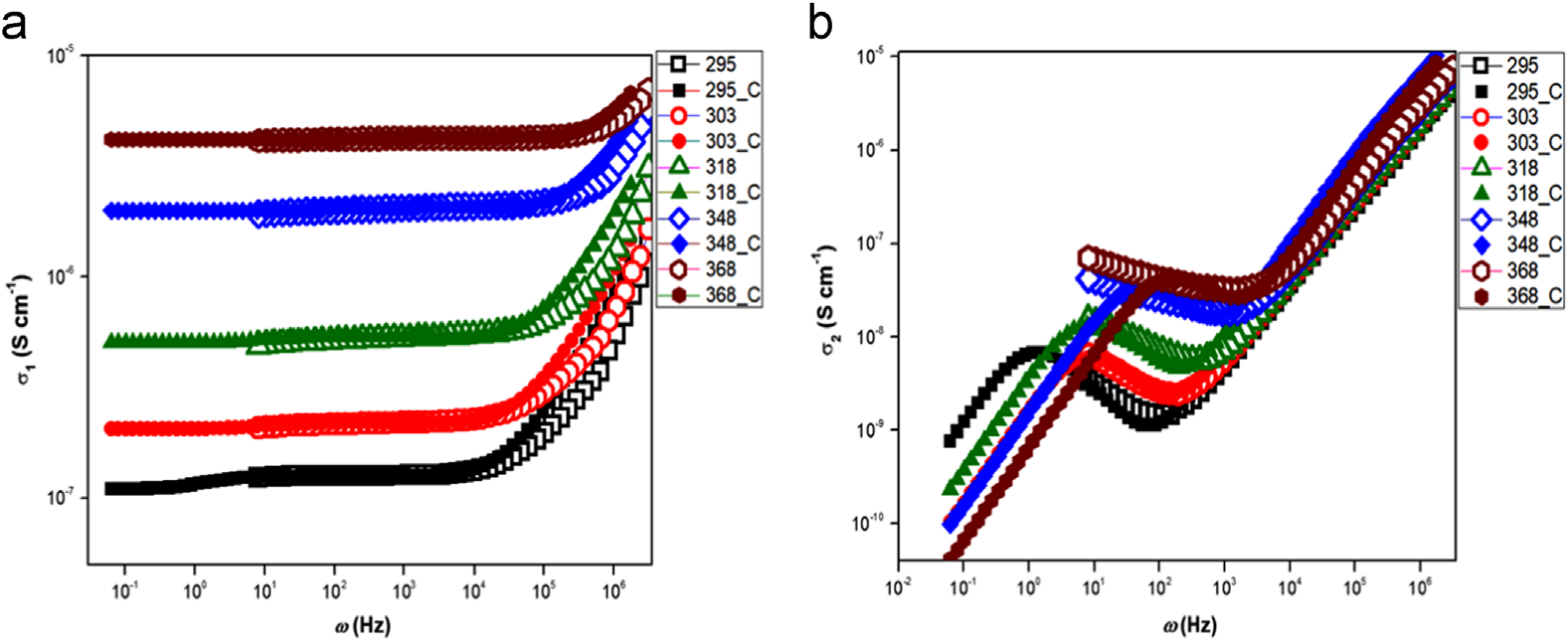
a) and **b)** are the real (σ_1_) and imaginary (σ_2_) part of conductivity response of Ag_15_ sample as a function of applied frequency at different temperatures between 295 K to 368 K. Note that, open symbols represent experimental data where as solid symbols represents random-walk model fitting.

Table 1 shows the actual composition and corresponding sample name/symbols used in the further analysis. Fig. 1 shows the image of the obtained glass samples after quenching and annealing. At the same time, disks shown in the same image, are pellets of the corresponding samples after polishing and before gold sputtering. Fig. 2 shows the nyquist plot obtained from impedance spectroscopy of the prepared series as a function of Ag concentrations at 368 K. Fig. 3 shows the real (*σ*_1_) and imaginary (*σ*_2_) parts of conductivities extracted using random-walk model from impedance data of Ag_15_ as a function of temperature. Fig. 3 represents the case example.

**Table 1:** Glass composition with its corresponding sample label.

*Sample denotation*	*Sample composition*
Ag_0_	Ge_16_Sb_12_Se_72_
Ag_0.2_	Ag_0.2_(Ge_16_Sb_12_Se_72_)_99.8_
Ag_0.4_	Ag_0.4_(Ge_16_Sb_12_Se_72_)_99.6_
Ag_0.6_	Ag_0.6_(Ge_16_Sb_12_Se_72_)_99.4_
Ag_0.8_	Ag_0.8_(Ge_16_Sb_12_Se_72_)_99.2_
Ag_1_	Ag_1_(Ge_16_Sb_12_Se_72_)_99_
Ag_5_	Ag_5_(Ge_16_Sb_12_Se_72_)_95_
Ag_10_	Ag_10_(Ge_16_Sb_12_Se_72_)_90_
Ag_15_	Ag_15_(Ge_16_Sb_12_Se_72_)_85_
Ag_20_	Ag_20_(Ge_16_Sb_12_Se_72_)_80_
Ag_25_	Ag_25_(Ge_16_Sb_12_Se_72_)_75_

## 2 Experimental design, materials, and methods

All of the samples, having nominal composition Ag_x_(Ge_16_Sb_12_Se_72_)_100-x_ with 0 ≤ *x* ≤ 25, were prepared by the melt quenching method. The exact composition and corresponding label is given in Table 1.

All of the elements (Ag, Ge, Sb, Se) used for the synthesis were of high purity 5 N. A batch of 10 g, with an appropriate amount of each element was mixed together to get a more homogeneous mixture and sealed in a quartz ampule (with the inner diameter of 10 mm) under high vacuum (10^-3^ Pa). The sealed ampule was loaded into a rocking tube furnace and heated with a rate 2 °C/min up to 950 °C before dwelling at 950 °C for 12 h in the rocking furnace. After 12 h, the temperature was slowly cooled down to 730 °C and at 730 °C ampules were quench into water, and later annealed at a temperature close to *T*_*g*_ for 4 h, followed by slow cooling down to room temperature. The obtained glass rods after annealing were shown in Fig. 1. For impedance measurements, the rods were cut into disks of 10 mm diameter and a thickness between 1–2 mm. The glass discs were polished with optical quality using SiC papers as well as Al_2_O_3_ powder with varying sizes such as 6 µm, 3 µm, and 1 µm. The obtained disks after polishing were also shown in Fig. 1.

For impedance measurements, the polished disks were sputtered with gold on both sides for a good electrical contact with the electrodes as well as to form a blocking electrode.

Impedance spectroscopy (IS) measurements were carried out on a PGSTAT 302 N with the FRA32 and NOVA 10 software. The cell used stainless disk-like electrodes sputtered with gold to block the Ag^+^ ions passage. Temperature dependence IS was carried out with an applied input voltage of 0.1 V, the temperature range from 298 K to 368 K, and a frequency range from 1 Hz to 50 kHz. The temperature was controlled with a Microcell HC set-up (Rhd instruments) with an accuracy of ± 1 °C. The input capacitance and impedance of the instrument were lower than 8 pF and higher than 100 GΩ, respectively. The obtained impedance data were first converted into its corresponding real (*ρ*_1_) and imaginary (*ρ*_2_) resistivity data to take account of sample dimensions using following relations,

$$\rho_{1}=Z_{1}A/L\;\mathrm{and}\;\rho_{2}=Z_{2}\;A/L.$$

The obtained values were plotted in the form of nyquist plot shown in Fig. 2 as a function of Ag concentrations. For better visibility, the data were splits into Fig. 2a and b. The data were further fitted using random-walk model [2] shown by solid lines in Fig. 2a and b.

To observe the temperature influence as well as to see the conductivity behavior, Ag15 were selected as a case example and impedance data were collected in the temperature range of 295 K to 368 K. The obtained impedance data were first converted to its corresponding resistivity using above relation. The obtained resistivity values were further converted into corresponding real (*σ*_1_) and imaginary (*σ*_2_) parts of conductivities using following relations.

$$\sigma_{1}=\frac{\rho_{1}}{\rho_{1}^{2}+\rho_{2}^{2}}\;\mathrm{and}\;\sigma_{2}=\frac{\rho_{2}}{\rho_{1}^{2}+\rho_{2}^{2}}$$

The obtained data were plotted in Fig. 3a and b as a function of applied frequency. Again to show the validity of random-walk model i.e. random-walk model can fit even in conductivity plot, the obtained conductivity plots were fitted using random-walk model shown by solid symbols in Fig. 3a and b.
