# Supplementary material for: Impedance spectroscopy data of Agx(Ge16Sb12Se72)100-x chalcogenide glasses
Source: Data Brief. 2019 Jan 18;22:1052–6. doi: 10.1016/j.dib.2019.01.025 (PMC6355960; doi:10.1016/j.dib.2019.01.025)
Supplement: Supplementary file 1 — Transparency document [file mmc1.docx]

January 03, 2019

To The Managing Editor -
Co-EiC
Data in Brief

Dear Editor,

Please see the attached file for an original manuscript for exclusive submission to the *Data in Brief* in the area of “Materials Science.” All authors have contributed to and have seen the manuscript and agree to its submission. The authors have no known conflicts-of-interest.

“*Impedance Spectroscopy data of Ag_x_(Ge_16_Sb_12_Se_72_)_100-x_ chalcogenide glasses*”

Deepak S. Patil (corresponding author), dspatil@nmhu.edu

Manisha Konale, st44439@student.upce.cz

Tomas Wagner, tomas.wagner@upce.cz

We believe that the data presented in the current draft is appropriate and of great interest to the ***Data in Brief*** readership, as it represent impedance data of solid state ionic conductor including Random-walk model fitting. We believe that the current data provides very useful information for the development of solid-state ionic batteries.

Please feel free to contact me if you require additional information.

Sincerely,

Deepak S. Patil

Research Associate,

PREM Fellow,

Department of Chemistry,

New Mexico Highlands University,

PO Box 9133

Las Vegas, NM 87701

Phone: +1 5054543035

[dspatil@nmhu.edu](mailto:dspatil@nmhu.edu)
